# CLINICAL CORRELATION OF MOCA AND TAU IMAGING

**DOI:** 10.1093/geroni/igad104.3228

**Published:** 2023-12-21

**Authors:** Surya Sunil, Malini Nair, Nicole Byrne, Adriana Rodriguez, Anil K Nair

**Affiliations:** Boston Neuropsychiatry, Boston, Massachusetts, United States; Boston Neuropsychiatry, Boston, Massachusetts, United States; Boston Neuropsychiatry, Boston, Massachusetts, United States; Boston Neuropsychiatry, Boston, Massachusetts, United States; Boston Neuropsychiatry, Boston, Massachusetts, United States

## Abstract

**Background:**

Tau-PET Scan measures brain tau deposition. Montreal Cognitive Assessment (MOCA) is used for cognitive impairment in (MCI) and Alzheimer Disease (AD). We analyzed the correlation of MOCA to tau-PET imaging in a memory clinic. Hypothesis Tau-PET positivity correlates with lower MOCA scores in memory clinic patients Objectives To assess the correlation of tau-PET positivity to MOCA test scores in memory clinic patients To evaluate an MOCA score cut-off that translates optimally to tau-PET positivity

**Method:**

Retrospective observational chart review study of 34 patients attending a memory clinic in Boston, MA, with MOCA score and tau-PET scan included for analysis No stratification based on initial or final diagnosis, severity of cognitive impairment, or tracer used. Baseline MOCA and a binary PET scan result were analyzed with covariates age, sex and race. Analysis done on ‘R’ v. 2.4.0. Pearson correlation estimated MOCA score to tau-PET status. Kruskal Wallis/Chi square tests for relationship of age, sex, gender and education with MOCA score and tau-PET status. ROC analyses and sensitivity/specificity were obtained.

**Results:**

Tau PET positivity did not correlate significantly with MOCA score (r= -0.24, 95%CI –0.54 to -0.12, p=0.19). A cut point of 22 on the MOCA estimated tau deposition in the brain by PET scan, with an AUC of 80%, 95% CI –0.6 to –0.99, possibly indicating a ceiling effect on the MOCA.

**Conclusion:**

Tau PET does not correlate with MOCA scores, possibly due to a ceiling effect in the mild impairment subjects.

